# Simulating the Dynamics of Scale-Free Networks via Optimization

**DOI:** 10.1371/journal.pone.0080783

**Published:** 2013-12-06

**Authors:** Tiago Alves Schieber, Martín Gómez Ravetti

**Affiliations:** Departamento de Engenharia de Produção, Universidade Federal de Minas Gerais, Belo Horizonte, Brazil; University of Warwick, United Kingdom

## Abstract

We deal here with the issue of complex network evolution. The analysis of topological evolution of complex networks plays a crucial role in predicting their future. While an impressive amount of work has been done on the issue, very little attention has been so far devoted to the investigation of how information theory quantifiers can be applied to characterize networks evolution. With the objective of dynamically capture the topological changes of a network's evolution, we propose a model able to quantify and reproduce several characteristics of a given network, by using the square root of the Jensen-Shannon divergence in combination with the mean degree and the clustering coefficient. To support our hypothesis, we test the model by copying the evolution of well-known models and real systems. The results show that the methodology was able to mimic the test-networks. By using this copycat model, the user is able to analyze the networks behavior over time, and also to conjecture about the main drivers of its evolution, also providing a framework to predict its evolution.

## Introduction

The idea of understanding the principles driving the evolution of complex networks has still a central place in the study of complex systems. Even with huge amounts of information available in the world, there are still many situations where access to high quality data with specific properties is very difficult and complicated. For example if we want to analyze the evolution of a gene-network during the progression of a disease, we will probably end with pieces of the puzzle, limited by the number of samples, or a small number of snapshots of the network over time. If we want to test an optimization algorithm to a specific network structure, like a power grid or telecom network, is it possible to generate instances with close-to-real characteristics, to help in the development and the efficiency test of the methodology?

With these problems in mind, the goal of our work is to introduce a novel model to analyze and reproduce complex network evolution. With this model we are able to compare different network dynamics, replicate them and even predict their behavior.

The first attempt to capture and study a network's evolution was due to Erdös and Rényi in 1959 [1] and it is sometimes called Classical Random Graph. The algorithm is based on a network with a fixed number of nodes then allows the connection of two of the nodes with probability 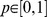. The result is a network with degree distribution taking a Poisson form.

In 1998 Watts and Strogatz [2] proposed a model that starting from a regular network lattice in wich each edge can be rewired to a randomly chosen vertex with a particular probability. After rewiring a small group of edges the network rapidly changes its topological properties as the average path length and its clustering coefficient. At each step of the process, the probability is increased and the network walks towards a random graph.

The first attempt to analyze the idea of growing networks presenting scale-free features, was addressed by Barabási and Albert [3] in 1999. They argued that the scale-free characteristic, corresponding to a power-law degree distribution, was originated by a concept known as preferential attachment, present in many real networks. Basically, it consists in the idea that nodes with higher degree tend to be more connected than nodes with lower degree values. They presented a model that at each step, a new node is included in the network and it connects to 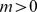 other nodes to the network with probability: 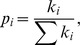 where, 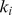 is the degree of the node 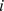 and the summation is over all nodes of the network. It can be proved that this network possess an average degree equals 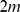 and a degree distribution following 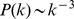 [4], [5].

The Barabási and Albert model can capture one mechanism that generates a power law distribution but, when modeling real networks, it has some drawbacks. For example, most real networks, exhibits different exponents on the power law distribution (see [6] for a deeper discussion on the topic) and the clustering coefficient of the generated network is usually lower than values in real situations. In 2000, Dorogotsev and Mendes [7] proposed an alteration on the preferential attachment equation. They ascertained that in some systems the probability of a connection is not only proportional to the node degree, but also depends on its age. Since then, several modifications on preferential attachment have been performed. In 2001, Barabasi *et al.* used direct measurements on the available data of the social network of scientific collaborations [8] and constructed a model that allows to investigate the large scale topology of the network and its dynamical features. After that, increasingly sophisticated models have been created (see [6] for deeper discussion on the topic).

In 2007, Goshal and Newman proposed a model that grows a network with any desirable degree distribution [9]. This model has some drawbacks because two networks with the same degree distribution are not necessarily isomorphic. Also in 2007, Leskovec, Kleinberg and Faloutsos created a model, called Forest Fire Model, based on the observation that densification power laws and shrinking effective diameters are properties that hold across a range of diverse networks [10]. In 2008, Leskovec, Backstrom, Kumar and Tomkins also created a model based on generation of triangles in the network (triangle closure) using the maximum-likelihood principle [11]. In 2010, Barthelemy proposed the tree growth model with local optimization [12] where spatial networks were considered. In 2011, Herrera and Zufiria proposed a model that creates networks with adjustable clustering coefficient via random walks [13]. In recent publications [14], [15], the discussion about the processes underlying the preferential attachment is revisited and a new model based on the popularity versus similarity was presented by Papadopoulos et al. [14]. One of the main questions behind this discussion is about the role of optimization in the organization and evolution of scale-free networks.

In [16] a methodology was proposed to study the evolution of small-world networks based on Information Theory quantifiers. Following that research approach, we proposed a model that use the square root of the Jensen-Shannon divergence embedded on an optimization algorithm to capture the evolution of scale-free networks. To test our algorithm three theoretical models are explored, the Barabasi-Albert (BA) [3], the Herrera-Zufiria (HZ) [13] and the popularity vs similarity model (PS) [14]. When considering real world networks, the Infectious Socio-Patterns dataset [17], the Online Forum Network [18], the UC Irvine message network [19] and the Hypertext 2009 dynamic contact network [17] are analyzed.

It is important to notice that our goal is to demonstrate that our model/methodology is able to capture and reproduce the growing process of a given network. With that objective in mind, we choose three theoretical models and four real networks, to prove how well our methodology performs. We are not suggesting the introduction of a theoretical model to mimic a real network. The comparison with theoretical models was performed to have a controlled experiment. The selected models are well known and studied.

## Methods

### Metrics

The degree of a node in a network is the number of edges incident to it. Given a network, we can define a probability distribution function (PDF) associated with the probability that a randomly selected node has degree 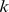. This PDF gives the spread in the number of edges that a node has. Many real networks exhibit a degree distribution following, at least asymptotically, a power law 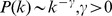 [4], [5].

Regarding the Information theory quantifiers, Shannon entropy measures the degree of heterogeneity of the network [20]. Its zero value corresponds to the state of having complete knowledge of the process (regular lattice). On the other hand, the maximum entropy value occurs when our knowledge of the system is minimized. The Shannon entropy of the degree distribution 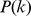 is defined by 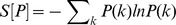.

The Jensen-Shannon divergence (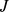) is a measure of the dissimilarity between two probability distributions. For two probability distributions 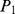 and 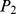, the 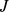 is given by, 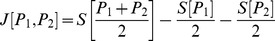. However, in order to obtain a real metric to quantify and compare states during a network evolution we use 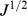, and fix as reference the uniform distribution (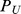) [21], [22]. The major importance in using the uniform distribution as reference is that it presents the biggest value for 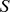 and there is no network that exhibits this degree distribution, thus, the network's value will never cross through the state characterized by this PDF as illustrates proposition 1.

**Proposition 1** *Given a network* 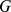 *with size* 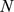 *and degree distribution* 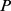 *then:*

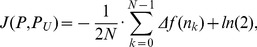
(1)where, 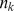 is the number of nodes with degree 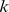 and

Readers can refer to File S1 or [16] for a deeper discussion on the topic.

Here we propose the use of the square root of Jensen-Shannon divergence to map the network evolution by the pair 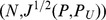. Although different networks could, by proposition 1, exhibit same values of the Jensen-Shannon divergence, it provides a remarkable help to characterize the network topology in the sense that it could measure the distances between degree distributions. Then, in order to capture the evolution of a network we need additional information: the average degree and the clustering coefficient.

One way to characterize the presence of triangles on the network is through the clustering coefficient (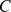). There are two different definitions of clustering coefficient: the first, also known as transitivity [23], is defined by:
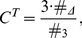
(3)


is the fraction of three times the number of triangles on the network by the number of connected triple (three nodes connected). The other one, is defined for each vertex of the network as:
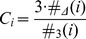



where, 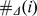 is the number of triangles involving vertex 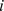 and 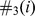 is the number of connected triples having i as the central vertex. Thus, the global clustering coefficient, 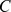, of the network is the average of 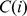 for every node 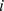:

### The Copycat Model

The model proposed here, from now on called the Copycat model (CP) reproduces dynamical changes in the topological properties, of a given network by considering the evolution of the distance between its degree distribution and the uniform distribution (reference).

By given the 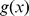, 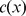, 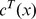 and 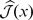 functions that respectively represent the mean degree, global clustering coefficient, transitive clustering coefficient and distance to the uniform distribution of a network with size 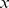, the algorithm 1 creates a new random network that preserve the values of these properties.

As input data, the algorithm also receives the initial network 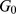, and the final number of nodes 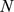 which is also the highest the network has in its evolution, considering the removal of nodes not allowed.

At each iteration one node is added and, using a simple computation procedure, it is decided how many links this new node will gain to maintain the mean average degree. To choose the nodes to which the new one is connected, the difference between the distance 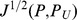 (new network to reference) and the distance 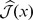 (copied network to reference), is minimized. The model chooses the node from a Restricted Candidate List (RCL), this procedure is similar to a classic heuristic procedure to solve combinatorial optimization problem called GRASP (Greedy Randomized Adaptive Search Procedure) [24]. As the algorithm randomly chooses a node to create the link, by controlling the random number generator seed it is possible to create as many different topologies which preserve the values of main properties, as desired. The Algorithm 1 gives details about the methodology. As it is possible to keep track of the degree distribution and by proposition 1, it is not expensive to recompute the square root of the Jensen-Shannon divergence.

**Figure 1:**
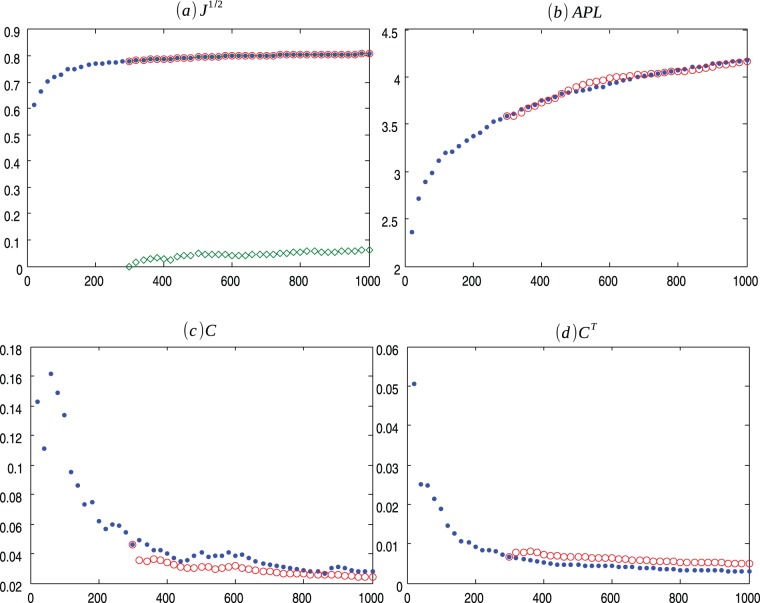
BA m = 2 evolution. Evolution of Barabasi-Albert (.) model for 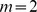 and the average values for 30 ensembles of Copycat model (o). (a) Square root of the Jensen-Shannon values (the 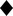 represents the mean of the square root of the Jensen-Shannon divergence values between the degree distribution generated by the CP model and the degree distribution of the BA network); (b) Average Path Length; (c) Clustering Coefficient; (d) Transient Clustering Coefficient. For exact values and confidence interval, see Table S1.

**Figure 2:**
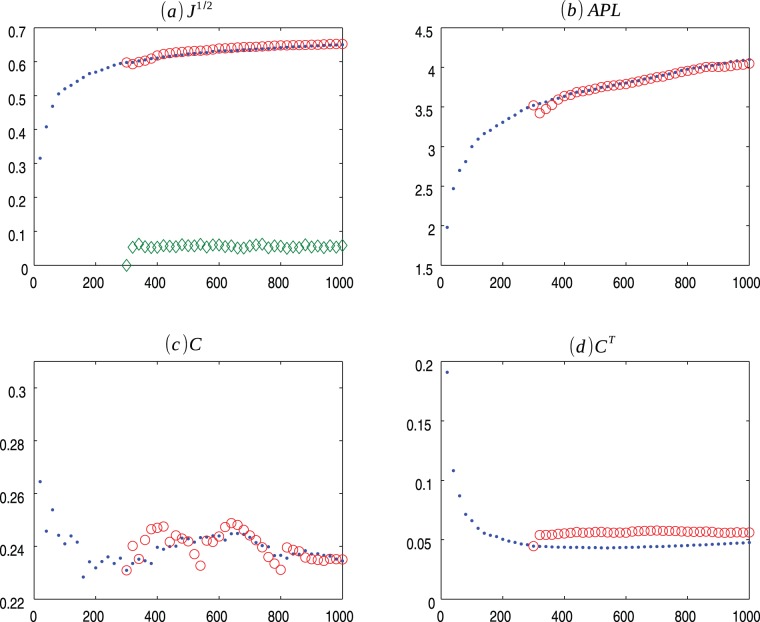
HZ p = 1 evolution. Evolution of Herrera-Zufiria (.) model for 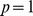 and the average values for 30 ensembles of Copycat model (o). (a) Square root of the Jensen-Shannon values (the 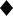 represents the mean of the square root of the Jensen-Shannon divergence values between the degree distribution generated by the CP model and the degree distribution of the HZ network); (b) Average Path Length; (c) Clustering Coefficient; (d) Transient Clustering Coefficient. For exact values and confidence interval, see Table S2.

**Figure 3:**
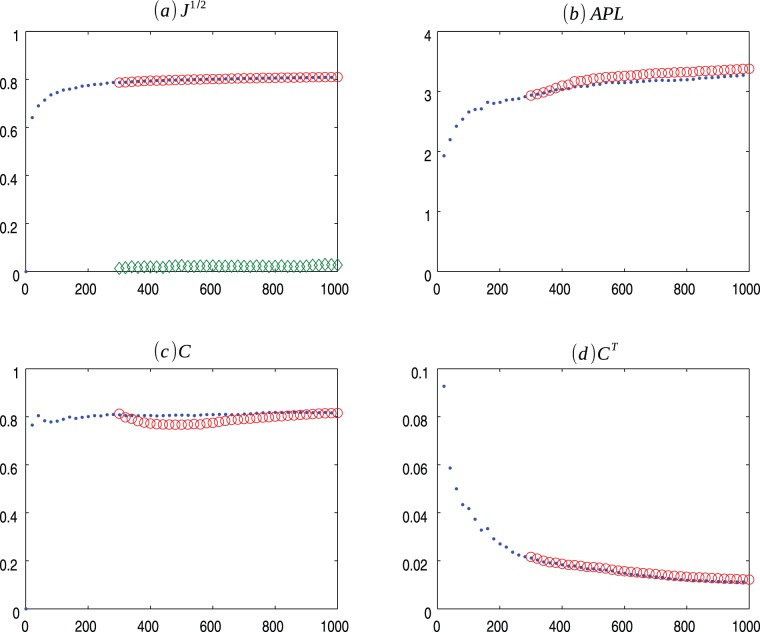
PS m = 2 evolution. Evolution of Popularity vs Similarity (.) model for 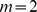 and the average values for 30 ensembles of Copycat model (o). (a) Square root of the Jensen-Shannon values (the 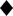 represents the mean of the square root of the Jensen-Shannon divergence values between the degree distribution generated by the CP model and the degree distribution of the PS network); (b) Average Path Length; (c) Clustering Coefficient; (d) Transient Clustering Coefficient. For exact values and confidence interval, see Table S3.

**Figure 4:**
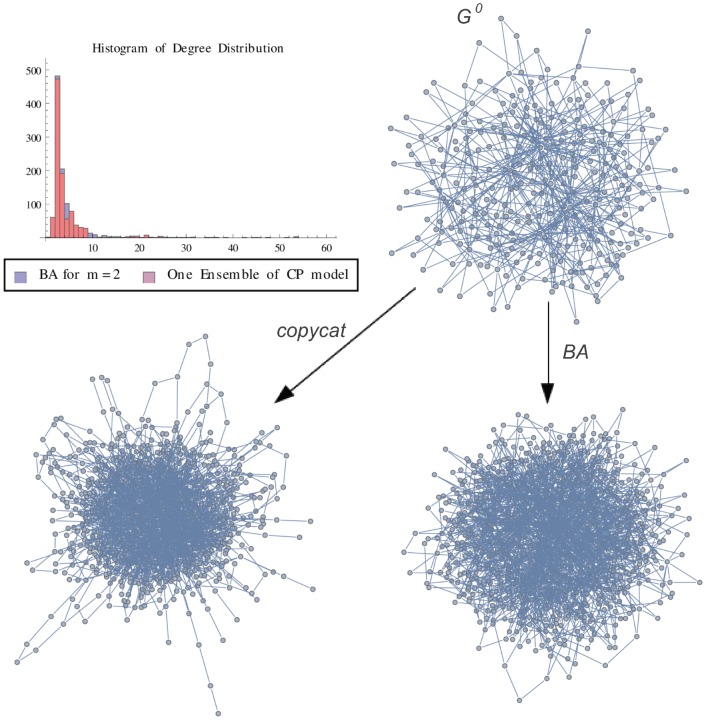
BA Graph vs One Ensemble. Graph representation of one ensemble of copycat model and the exact result for Barabasi-Albert Graph (m = 2). The figure shows the histogram of degree distributions of BA and CP networks. 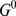 is the initial graph of CP model.

**Figure 5:**
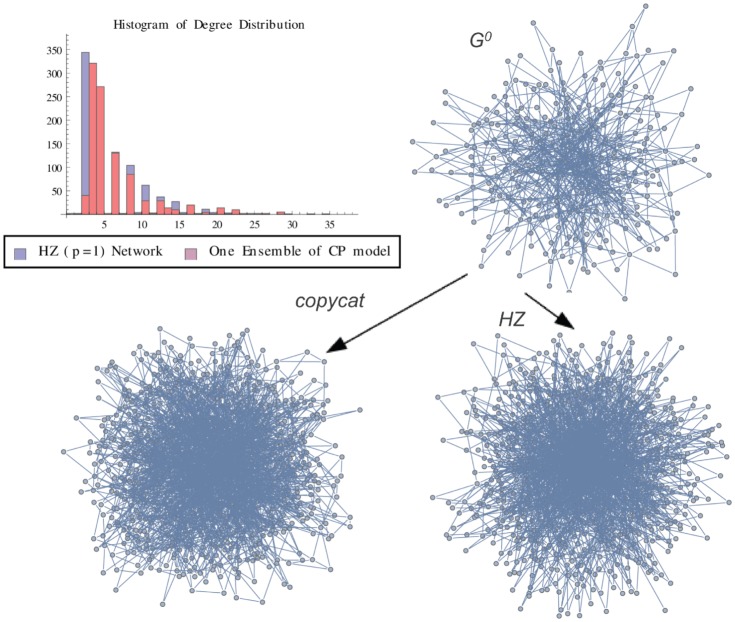
HZ Graph vs One Ensemble. Graph representation of one ensemble of copycat model and the exact result for Herrera-Zufiria Graph (p = 1). The figure shows the histogram of degree distributions of HZ and CP networks. 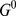 is the initial graph of CP model.

**Figure 6:**
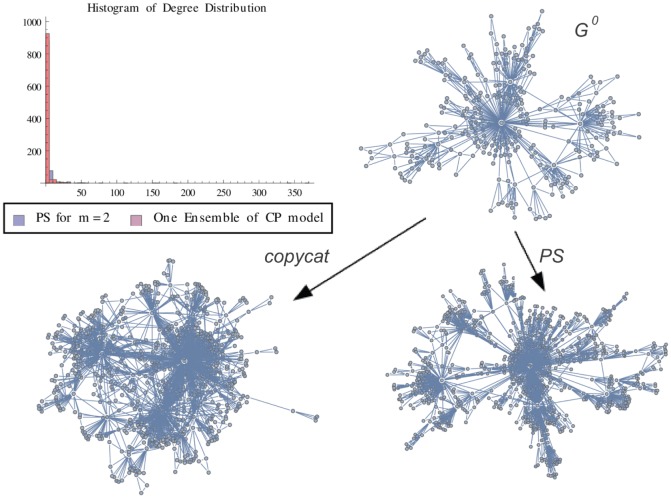
PS Graph vs One Ensemble. Graph representation of one ensemble of copycat model and the exact result for Popularity vs Similarity Graph (m = 2). The figure shows the histogram of degree distributions of PS and CP networks. 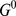 is the initial graph of CP model.

**Figure 7:**
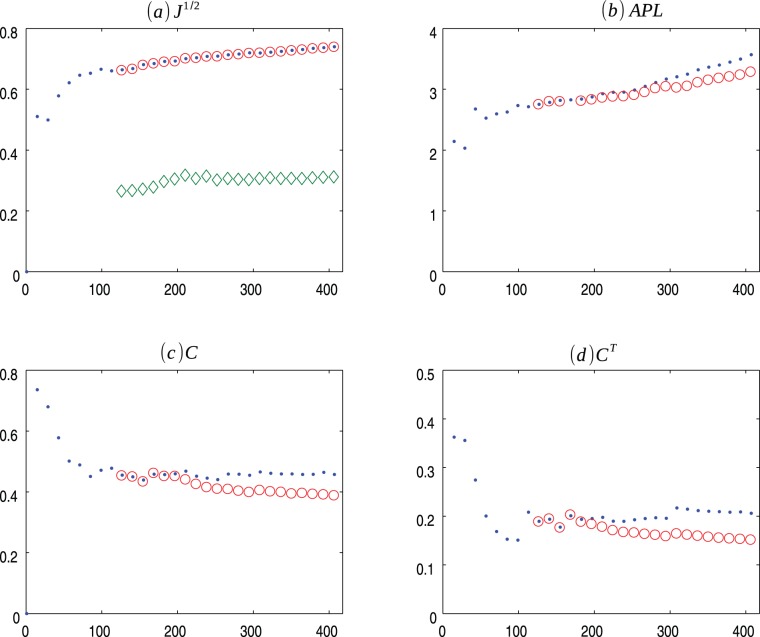
IS Evolution. Evolution of Infectious Socio-Patterns (.) network and the average values for 30 ensembles of Copycat model (o). (a) Square root of the Jensen-Shannon values (the 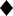 represents the mean of the square root of the Jensen-Shannon divergence values between the degree distribution generated by the CP model and the degree distribution of the IS network); (b) Average Path Length; (c) Clustering Coefficient; (d) Transient Clustering Coefficient. For exact values and confidence interval, see Table S4.

**Figure 8:**
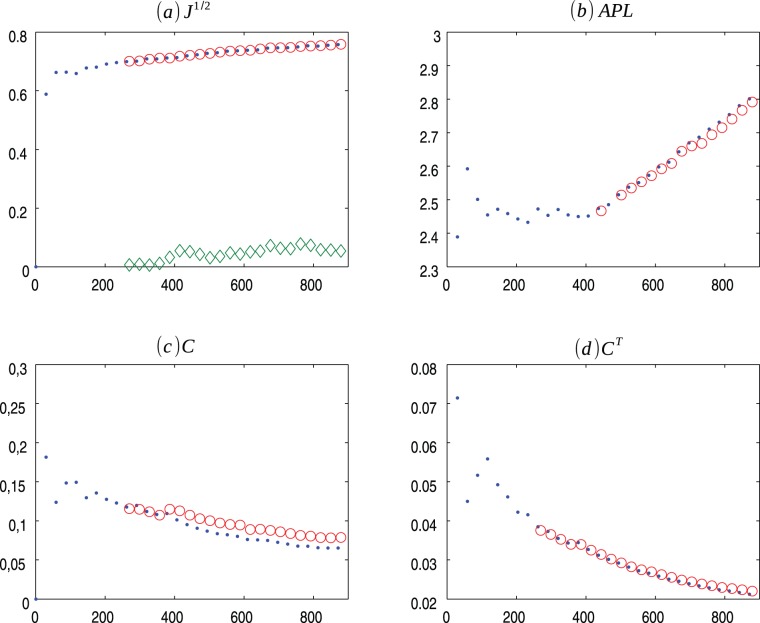
OF Evolution. Evolution of Online Forum (.) network and the average values for 30 ensembles of Copycat model (o). (a) Square root of the Jensen-Shannon values (the 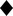 represents the mean of the square root of the Jensen-Shannon divergence values between the degree distribution generated by the CP model and the degree distribution of the OF network); (b) Average Path Length; (c) Clustering Coefficient; (d) Transient Clustering Coefficient. For exact values and confidence interval, see Table S5.

**Figure 9:**
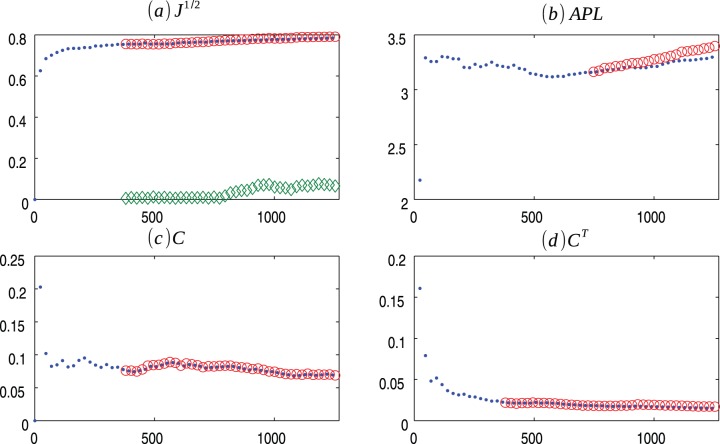
UCI Evolution. Evolution of UC Irvine messages (.) network and the average values for 30 ensembles of Copycat model (o). (a) Square root of the Jensen-Shannon values (the 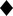 represents the mean of the square root of the Jensen-Shannon divergence values between the degree distribution generated by the CP model and the degree distribution of the UCI network); (b) Average Path Length; (c) Clustering Coefficient; (d) Transient Clustering Coefficient. For exact values and confidence interval, see Table S6.

**Figure 10:**
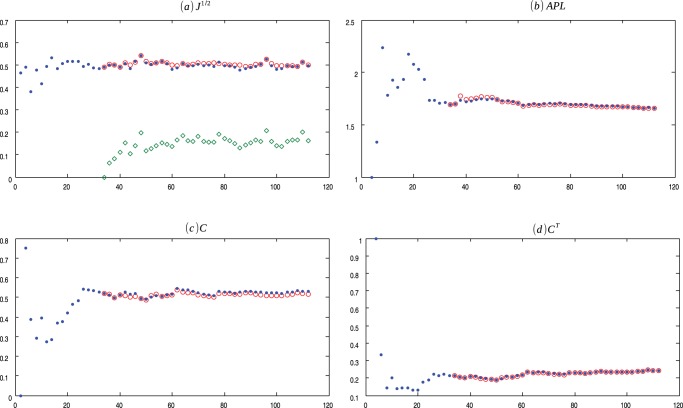
HT evolution. Evolution of Hypertext (.) network for and the average values for 30 ensembles of Copycat model (o). (a) Square root of the Jensen-Shannon values (the 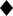 represents the mean of the square root of the Jensen-Shannon divergence values between the degree distribution generated by the CP model and the degree distribution of the HT network); (b) Average Path Length; (c) Clustering Coefficient; (d) Transient Clustering Coefficient. For exact values and confidence interval, see Table S7.

**Figure 11:**
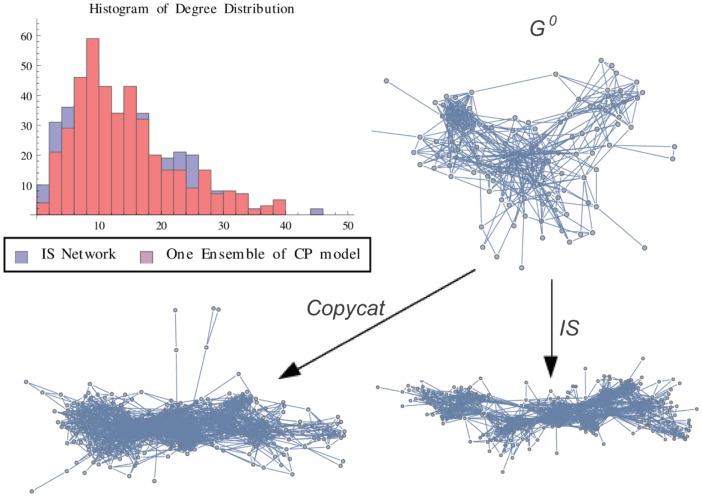
IS Graph vs One Ensemble. Graph representation of one ensemble of copycat model and the exact result for IS network. The figure shows the histogram of degree distributions of IS and CP networks. 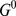 is the initial graph of CP model.

**Figure 12:**
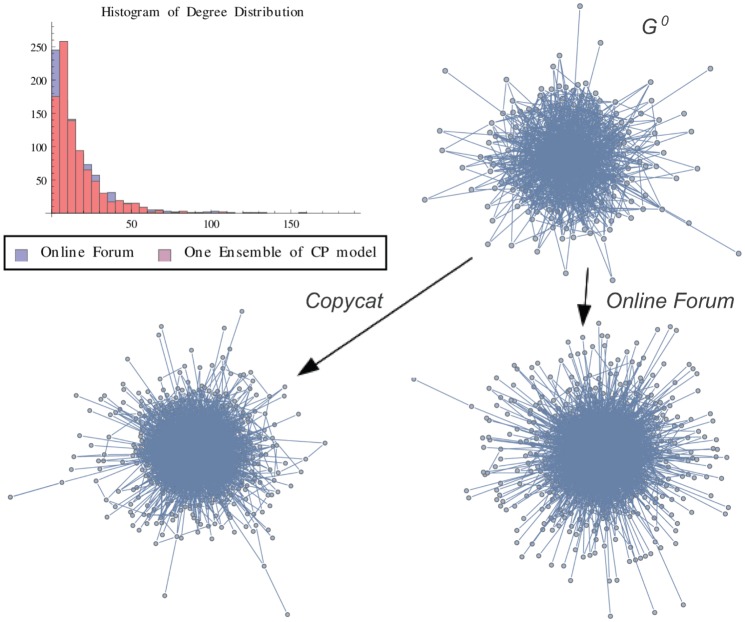
OF Graph vs One Ensemble. Graph representation of one ensemble of copycat model and the exact result for OF network. The figure shows the histogram of degree distributions of OF and CP networks. 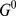 is the initial graph of CP model.

**Figure 13:**
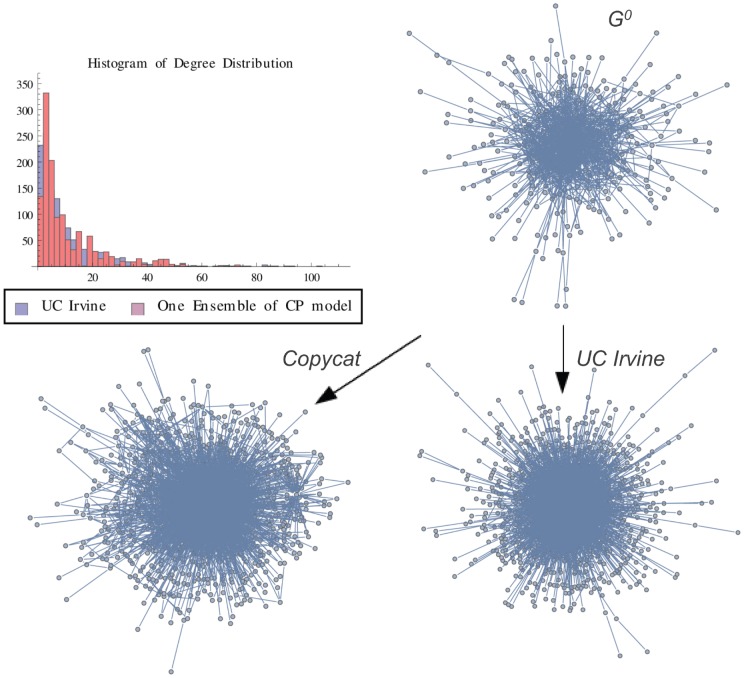
UCI Graph vs One Ensemble. Graph representation of one ensemble of copycat model and the exact result for IS network. The figure shows the histogram of degree distributions of IS and CP networks. 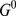 is the initial graph of CP model.

**Figure 14:**
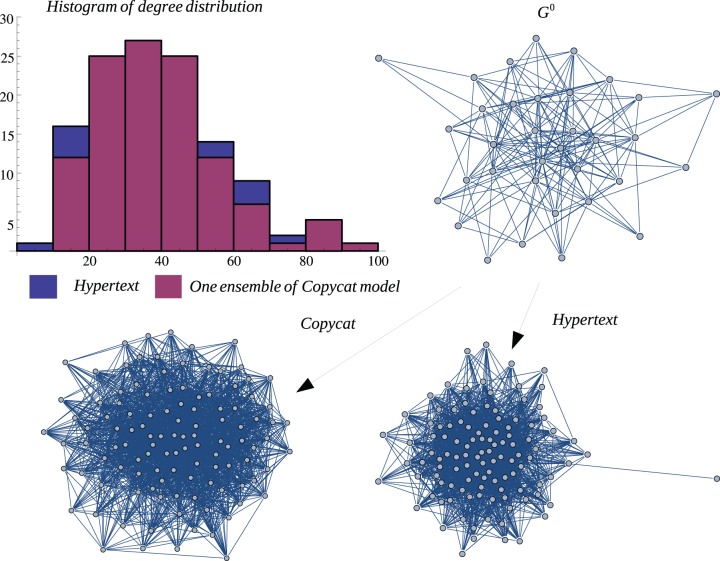
HT Graph vs One Ensemble. Graph representation of one ensemble of copycat model and the exact result for HT network. The figure shows the histogram of degree distributions of HT and CP networks. 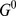 is the initial graph of CP model.

After getting the information about the three theoretical models, the Copycat Model was able to simulate their evolution, maintaining complex network properties values. This type of model opens a huge range of possibilities. It is possible to create specific functions (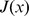) to simulate changes in the system's evolution, simulate an sporadic event, and even create several network instances to test special algorithms or approaches. Figures 1, 2, 3, 4, 5, 6, 7, 8, 9, 10, 11, 12, 13, 14 and Tables S1, S2, S3, S4, S5, S6, S7 depict how the CP replicates the evolution of studied models for a given set of parameters. It is noticeable that some networks characteristics from the original networks remain in a close range by the model's network, without using this information in the copycat procedure.

**Data**: 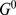, 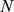, 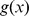, 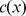, 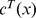 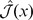


**Result**: Network topology with 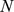 nodes and the desired metric values

**for** *i* = 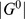 to 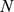 **do**

Compute the mean degree of the network, (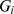);

Add a new node;
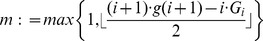



**while** 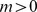 **do**

Create a list of candidates (RLC) with of nodes 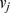, such that if a connection between 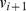 and 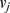 is performed, the difference between the distances of their degree distributions to the reference is minimized

Randomly choose a node from RCL

m = m-1

Compute the clusterings coefficients of the resulting network (*C*_*i*+1_ and );


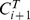
  Define 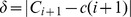 and 
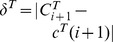
;

**if** 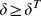 **then**


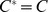
 and 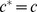


**end**

**if** 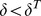 **then**


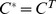
 and 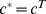


**end**

**if** 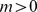 and 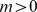 **then**

Create a list of candidates (RLC) with of nodes 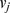 such that the distance

between 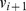 and 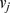 equals 2, and the clustering coefficient of the resulting

network, after connection 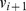 and 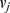 is performed, possess its maximum

value;

Randomly choose a node from RCL;

m = m-1

**end**

**end**

**end**

**Algorithm 1.** Pseudo-code of the Copycat Model. The algorithm receives as input parameters, an initial graph, the number of nodes, the mean degree (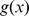), the average clustering coefficient (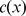), the transitive clustering coefficient (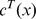) and its distance to the reference to copy the evolution of the network (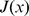). It is important to notice that the fastest convergence of the mean degree and the average clustering coefficient to specific values, allows us to use constant values instead of functions when analyzing bigger networks.

## Results and Discussion

Here we simulate the copycat model for three artificial networks (BA, HZ and PS models) and four real networks: Infectious Socio-Patterns (IS), Online Forum Network (OF), UC Irvine messages network (UCI) and Hypertext 2009 contact network (HT). For each network tested, the copycat model was run 30 times independently (different seeds). These ensembles were generated considering as the initial graph, 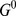, as the graph obtained by the evolution network until 30% of its final size: for example, for the BA graph we consider 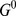 as the graph obtained by the model until size of 300 and then we copy its evolution until the size of 1000. We then plot the average of the square root of Jensen-Shannon Divergence values (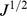), average path length (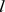 – see [23] for more details), global clustering coefficient (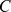) and transitive clustering coefficient (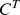) as networks evolves over time and the average of the result of the 30 ensembles of Copycat model approximation (Figures 1, 2, 3, 4, 5, 6, 7, 8, 9, 10, 11, 12, 13, 14). Moreover, we consider other networks characteristics: centrality measures [25], diameter, average neighbor degree, graph energy, spectrum, link density (see File S1 for more information) comparing the average of final results of the 30 ensembles of copycat model and the real values obtained in the network evolution. For every metric analyzed the distribution of the computed values presented a symmetric shape, thus we compute the confidence interval for 95% of confidence, using the Student's t-distribution (Tables S1, S2, S3, S4, S5, S6, S7).

### Artificial Networks

Figures 1, 2, 3 presents the comparisons between BA (m = 2), HZ (p = 1) and PS (m = 2) with copycat model evolution. It is important to notice that in all three cases, the copycat model considerably fits the square root of Jensen Shannon Divergence values, average path length, and clustering coefficient values of these artificial networks. Tables S1, S2, S3 depict the other network quantifiers computed.

In order to illustrate a possible result of the copycat model, Figures 4, 5, 6 show the generation of one ensemble of copycat model and comparisons between the desirable network and the histogram of degree distributions.

### Real Systems

The **Infectious Socio-Patterns dataset (IS)** contains the daily cumulated networks represented in the Infectious Socio-Patterns visualization [17]. The nodes represent visitors of the Science Gallery while the edges represent close-range face-to-face proximity between the concerned persons for each of the sixty-nine covered days. The network is undirected and we consider it unweighted. For convenience, we have chosen the day with the higher number of nodes (417 nodes) and the time evolution was considered by the increasing order of the node id. The data are distributed to the public under a Creative Commons Attribution-NonCommercial-ShareAlike license (http://creativecommons.org/licenses/by-nc-sa/3.0/) and can be found at http://www.sociopatterns.org.

The **Online Forum Network network (OF)** represents not the private messages exchanged among users, but the users' activities in the forum [18]. The forum represents an interesting two-mode network among 899 users and 522 topics in which a weight can be assigned to the ties based on the number of messages or characters that a user posted to a topic. The two-mode networks are projected onto one-mode networks using the procedure outlined on the projecting two-mode networks onto weighted one-mode networks-page (http://toreopsahl.com/tnet/two-mode-networks/projection/) and analyzed as an undirected network. Again the arrival time of edges is known, thus, it is possible to reconstruct the state of the network at any previous point in time. The data are freely distributed to the public through the webpage http://toreopsahl.com/datasets/#online_forum_network.

The **UC Irvine messages network (UCI)**, part of the Koblenz Network Collection, is the the Facebook-like Social Network originate from an online community for students at University of California, Irvine [19]. The original dataset includes the users that sent or received at least one message (1,899). For simplicity, for each of the directed graphs, we create their undirected counterparts by taking into account only bi-directional links between the users resulting in an undirected network with 1,265 nodes. The arrival time of edges is known, then, it is possible to reconstruct the state of the network at any previous point in time. The data are freely distributed to the public through the webpage http://toreopsahl.com/datasets/#online_forum_network.

The **Hypertext 2009 dynamic contact network (HT)** the dynamical network of face-to-face proximity of 113 conference attendees over about 2.5 days [17]. The nodes represent a person while the edges represent a contact of, at least, 20 seconds between two persons. The network is undirected and we consider it unweighted. For convenience, the time evolution is considered by the increasing order of the node id. The data are distributed to the public under a Creative Commons Attribution-NonCommercial-ShareAlike license (http://creativecommons.org/licenses/by-nc-sa/3.0/) and can be found at http://www.sociopatterns.org.

After getting the information about the three real networks, the Copycat model was able to simulate their evolution, maintaining some complex network properties values.

Figures 7, 8, 9, 10 presents the comparisons between IS, OF, UCI and HT with copycat model evolution. Moreover, Tables S4, S5, S6, S7 depict other network quantifiers values.

In order to illustrate a possible result, Figures 10, 11, 12 show the generation of one ensemble of copycat model and the graphical comparisons of final result of histogram of degree distributions.

### Final Remarks

In this article we propose a novel methodology to capture the dynamic behavior of scale-free networks. The methodology is based on Information Theory quantifiers, that, when embedded in an optimization algorithm creates a model able to reproduce the behavior of networks' evolution. The main difference against other models is its ability to capture oscillations during its evolution. Most models must be previously adjusted to create a network with fixed properties. The proposed model has the ability to dynamically adjust the topological properties step by step during the network's creation. Its main drawback when compared with the abovementioned models is that, the decisions at each node inclusion are computationally more expensive: at each node inclusion the model has to solve an optimization problem, that consists in determining how many and which links are necessary to reach the stage of the copied network. To improve its computational time, a heuristic-like procedure was included.

By using this copycat model, the user is able to analyze the network's behavior over time, and also to conjecture about the main drivers of its evolution. Last but not least, it provides a framework to predict its evolution.

## Supporting Information

File S1 **Contains the proof of Proposition 1 and measurements on complex networks.**
(PDF)

Table S1 **Comparisons of the desired value and confidence interval for 30 copycat results to other measures on complex networks for the Barabasi-Albert Graph with m = 2.**
(TIF)

Table S2 **Comparisons of the desired value and confidence interval for 30 copycat results to other measures on complex networks for the Herrera-Zufiria Graph with p = 1.**
(TIF)

Table S3 **Comparisons of the desired value and confidence interval for 30 copycat results to other measures on complex networks for the Popularity-Similarity Graph with m = 2.**
(TIF)

Table S4 **Comparisons of the desired value and confidence interval for 30 copycat results to other measures on complex networks for the Infectious SocioPatterns Graph.**
(TIF)

Table S5 **Comparisons of the desired value and confidence interval for 30 copycat results to other measures on complex networks for the Online Forum Network Graph.**
(TIF)

Table S6 **Comparisons of the desired value and confidence interval for 30 copycat results to other measures on complex networks for the UC Irvine Network Graph.**
(TIF)

Table S7 **Comparisons of the desired value and confidence interval for 30 copycat results to other measures on complex networks for the HT network.**
(TIF)
