# Supplementary material for: Simulating the Dynamics of Scale-Free Networks via Optimization
Source: PLoS One. 2013 Dec 6;8(12):e80783. doi: 10.1371/journal.pone.0080783 (PMC3865918; doi:10.1371/journal.pone.0080783)
Supplement: File S1 — Contains the proof of Proposition 1 and measurements on complex networks. (PDF) [file pone.0080783.s001.pdf]

Simulating the dynamics of scale-free networks via optimization - Supporting Information

Tiago Alves Schieber[1], Martín Gómez Ravetti[1,*]

[1] Departamento de Engenharia de Produção, Universidade Federal de Minas Gerais, Belo Horizonte, Brazil

* E-mail: tiagoschieber@eng-pro.dout.ufmg.br, martin.ravetti@dep.ufmg.br

Proof and Consequences of Proposition 1

Proposition 1. *Given a network G with size N and degree distribution P then:*

$$\mathcal{J}(P, P_U) = -\frac{1}{2N} \cdot \sum_{k=0}^{N-1} \Delta f(n_k) + ln(2), \tag{1}$$

where, n_k is the number of nodes with degree k and

$$f(x) = \begin{cases} 0, & \text{if } x \leq 0 \\ x \cdot ln(x), & \text{otherwise} \end{cases} \qquad \text{and} \qquad \Delta f(x) = f(x+1) - f(x). \tag{2}$$

Proof: By definition:

$$\mathcal{J}(P, P_U) = S\left[\frac{P + P_U}{2}\right] - \frac{S[P] + S[P_U]}{2}. \tag{3}$$

Defining n_k as the number of nodes with degree k, then:

$$P(k) = \frac{n_k}{N} \qquad \text{and} \qquad P_U(k) = \frac{1}{N} \qquad \forall\, k = 0,\ 1,\ 2 \cdots N-1.$$

The first term on the right hand side of equation (3) is given by

$$\begin{aligned} S\left[\frac{P + P_U}{2}\right] &= -\sum_{k=0}^{N-1} \frac{n_k + 1}{2N} \cdot ln\left(\frac{n_k + 1}{2N}\right) \\ &= -\sum_{k=0}^{N-1} \frac{n_k + 1}{2N} \cdot ln\,(n_k + 1) + \sum_{k=0}^{N-1} \frac{n_k + 1}{2N} \cdot ln\,(2N) \\ &= -\frac{1}{2N} \sum_{k=0}^{N-1} (n_k + 1) \cdot ln\,(n_k + 1) + ln(2N), \end{aligned} \tag{4}$$

where, in the last equality, we use the fact that $\sum_{k=0}^{N-1} n_k = N$.

The second term on the right hand side of equation (3) is given by

$$\begin{aligned} -\frac{S[P] + S[P_U]}{2} &= \frac{1}{2N}\left[\sum_{k=0}^{N-1} n_k \cdot ln\left(\frac{n_k}{N}\right) + \sum_{k=0}^{N-1} ln\left(\frac{1}{N}\right)\right] \\ &= \frac{1}{2N}\left[\sum_{k=0}^{N-1} n_k \cdot ln\,(n_k) - \sum_{k=0}^{N-1} n_k \cdot ln(N) + \sum_{k=0}^{N-1} ln\left(\frac{1}{N}\right)\right] \\ &= \frac{1}{2N} \sum_{k=0}^{N-1} (n_k) \cdot ln\,(n_k) - ln(N), \end{aligned} \tag{5}$$

where, in the last equality, we use the fact that $\sum_{k=0}^{N-1} n_k = N$.

Combining equations (2), (4) and (5) we obtain the result.

□

Corollary 1. *Given a network G with size N and degree distribution P then:*

$$\mathcal{J}(P, P_U) < ln(2).$$

Proof: observe that $f(n_k+1) - f(n_k) \geq 0$ for all $n_k = 0,\ 1,\ 2\cdots$. Then, using the fact that in a network of size N, $\sum_{k=0}^{N-1} n_k = N$, we have:

$$\sum_{k=0}^{N-1} \Delta f(n_k) > 0. \tag{6}$$

Thus, by equation (6) and Proposition 1 we obtain the result.

□

Proposition 1 offers an important tool in analysis of complex networks: if a new connection in a network is made, at most four terms on the right hand side of equation (1) should be computed. In other words, adding a new connection from a node with degree k_1 to a node with degree k_2:

$$n_{k_1} \to n_{k_1} - 1, \qquad n_{k_1+1} \to n_{k_1+1} + 1, \qquad n_{k_2} \to n_{k_2} - 1, \qquad n_{k_2+1} \to n_{k_2+1} + 1,$$

then, to compute changes in the Jensen-Shannon divergence value of the network when a new connection is made, we do not need to look up all degree distribution of the network.

Another consequence of this proposition stays in the fact that two different networks could exhibit the same Jensen-Shannon divergence value.

Measurements on complex networks

To validate our approach, we measure another properties of the network: related with distance (diameter and average path length), with degree distribution (link density, square root of the Jensen-Shannon divergence values and average neighbor degree), with clustering and cycles (clustering coefficient, transitive clustering coefficient and number of loops), with centrality (betweenness centrality, eigenvector centrality and closeness centrality) and spectral measurements (graph energy, graph spectrum and S-metric). Here we present a brief definition of each measure for an undirected and unweighted network G with size N and adjacency matrix A. Readers can refer to [1, 2] for a deeper discussion on the topic.

Measurements related with distance

The distance, $d(i, j)$ between the nodes i and j is defined as the minimum number of edges connecting them. The *average path length* (l) is the average of all possible distances in the network. Thus, for an undirected and unweighted network:

$$l = \frac{\sum_{i,j} d(i,j)}{N \cdot (N-1)}.$$

The *diameter* of a network is the maximum distance of any two nodes on the network:

$$D = \max_{i,j}\{d(i,j)\ \ \forall\, i,\ j\}$$

Measurements related with degree distribution

The *square root of Jensen-Shannon divergence values* is well defined in the main body of the paper. The *link density* of a network is the fraction between the total number of edges and $N \cdot (N-1)/2$ (the total number of possible edges). The *average neighbor degree* computes the average degree of neighboring nodes for every vertex.

Measurements related with clustering and cycles

The *clustering coefficient*, C, and the *transitive clustering coefficient*, C^T, are well defined in the main body of the paper. The number of loops in a network G is the number of edges that need to be removed in order that the graph cannot have cycles.

Centrality measures

The *betweenness centrality* measure of a node u quantifies its importance in terms of interactions of nodes on the network. It is defined by:

$$\sum_{i,j} \frac{\sigma(i,u,j)}{\sigma(i,j)}$$

where, $\sigma(i,u,j)$ is the number of shortest paths between i and j passing through u and $\sigma(i,j)$ is the number of shortest paths between i and j.

The *closeness centrality* measure of a node i is given by:

$$\frac{1}{\sum_j d(i,j)}$$

It can be viewed as the efficiency of each vertex (individual) in spreading information to all other vertices. The larger the closeness centrality of a vertex, the shorter is the average distance from the vertex to any other vertex, and thus the better positioned the vertex is in spreading information to other vertices.

Let X be a normalized eigenvector with respect to the greater eigenvalue λ of the adjacency matrix A, we define the *eigenvector centrality* of a node i, simply by $X(i)$.

Spectral measurements

The *graph energy* is, by definition, the sum of absolute values of the eigenvalues of A. The *graph spectrum* is defined as the eigenvalues of the Laplacian of the graph.

References

1. L. Rodrigues, G. Travieso, and P. R. Villas Boas. Characterization of complex networks: A survey of measurements. *Advances in Physics*, 56(1):167–242, August 2006.

2. Swami Iyer, Timothy Killingback, Bala Sundaram, and Zhen Wang. Attack robustness and centrality of complex networks. *PLoS ONE*, 8:e59613, 2013.
